# The Era Key to U. S. P., 1893

**Published:** 1893-10

**Authors:** 


THE ERA KEY TO THE U. S. P., 1893. Published by D. O. Haynes & Co., P. O. Box 583, Detroit, Michigan. Price, 25 cents.

The object of this work, as explained by the publishers, is to assist physicians and pharmacists to familiarize themselves with the contents of the new

*United States Pharmacopœia,* also to further the introduction and employment of official drugs and preparations.

This book gives in a very condensed form all the vital information regarding the drugs and preparations of the new *Pharmacopœia* as follows:

1. A complete *list* of all drugs and preparations in the new *U. S. P.* 2. The common *names* and *synonyms* of each drug and preparation. 3. The *parts* employed. 4. The *doses* in both Apothecaries and Metric Systems. 5. The *preparations* in which the drug is employed.

Particular attention has been paid to the typographical arrangement of the matter. The official names are arranged alphabetically in black-faced type, and no less than six styles of type are used in its composition so as to bring out in marked contrast the important features. The book is in vest-pocket size, and certainly of great assistance to physicians in writing prescriptions, and to pharmacists in dispensing the same.

